# The QardioArm App in the Assessment of Blood Pressure and Heart Rate: Reliability and Validity Study

**DOI:** 10.2196/mhealth.8458

**Published:** 2017-12-15

**Authors:** Victoria Mazoteras Pardo, Marta E Losa Iglesias, José López Chicharro, Ricardo Becerro de Bengoa Vallejo

**Affiliations:** ^1^ Universidad Complutense de Madrid Madrid Spain; ^2^ Universidad Rey Juan Carlos Alcorcon Spain

**Keywords:** blood pressure, heart rate, reliability, validity, mobile apps

## Abstract

**Background:**

Self-measurement of blood pressure is a priority strategy for managing blood pressure.

**Objective:**

The aim of this study was to evaluate the reliability and validity of blood pressure and heart rate following the European Society of Hypertension’s international validation protocol, as measured with the QardioArm, a fully automatic, noninvasive wireless blood pressure monitor and mobile app.

**Methods:**

A total of 100 healthy volunteers older than 25 years from the general population of Ciudad Real, Spain, participated in a test-retest validation study with two measurement sessions separated by 5 to 7 days. In each measurement session, seven systolic blood pressure, diastolic blood pressure, and heart rate assessments were taken, alternating between the two devices. The test device was the QardioArm and the previously validated criterion device was the Omron M3. Sessions took place at a single study site with an evaluation room that was maintained at an appropriate temperature and kept free from noises and distractions.

**Results:**

The QardioArm displayed very consistent readings both within and across sessions (intraclass correlation coefficients=0.80-0.95, standard errors of measurement=2.5-5.4). The QardioArm measurements corresponded closely to those from the criterion device (r>.96) and mean values for the two devices were nearly identical. The QardioArm easily passed all validation standards set by the European Society of Hypertension International Protocol.

**Conclusions:**

The QardioArm mobile app has validity and it can be used free of major measurement error.

## Introduction

Blood pressure (BP) plays a major role in the development of cardiovascular disease, which is the leading cause of premature death worldwide [1]. For patients who have or are at risk for high BP, regular monitoring of BP is useful [1,2].

Successful BP management depends to a large extent on the patient’s willingness and capacity to make certain lifestyle changes [3]. As demonstrated in studies by the UK National Health System, self-measurement of BP is a priority strategy for managing BP [4]. Self-measured BP monitoring has many advantages. It enables diagnosis of hypertension or hypotension, helps patients control BP, improves therapeutic compliance, and minimizes the “white coat” and “masked hypertension” syndromes and observation biases caused by the health care professional being aware of the patient’s characteristics during the measurement [5-8].

**Figure 1:**
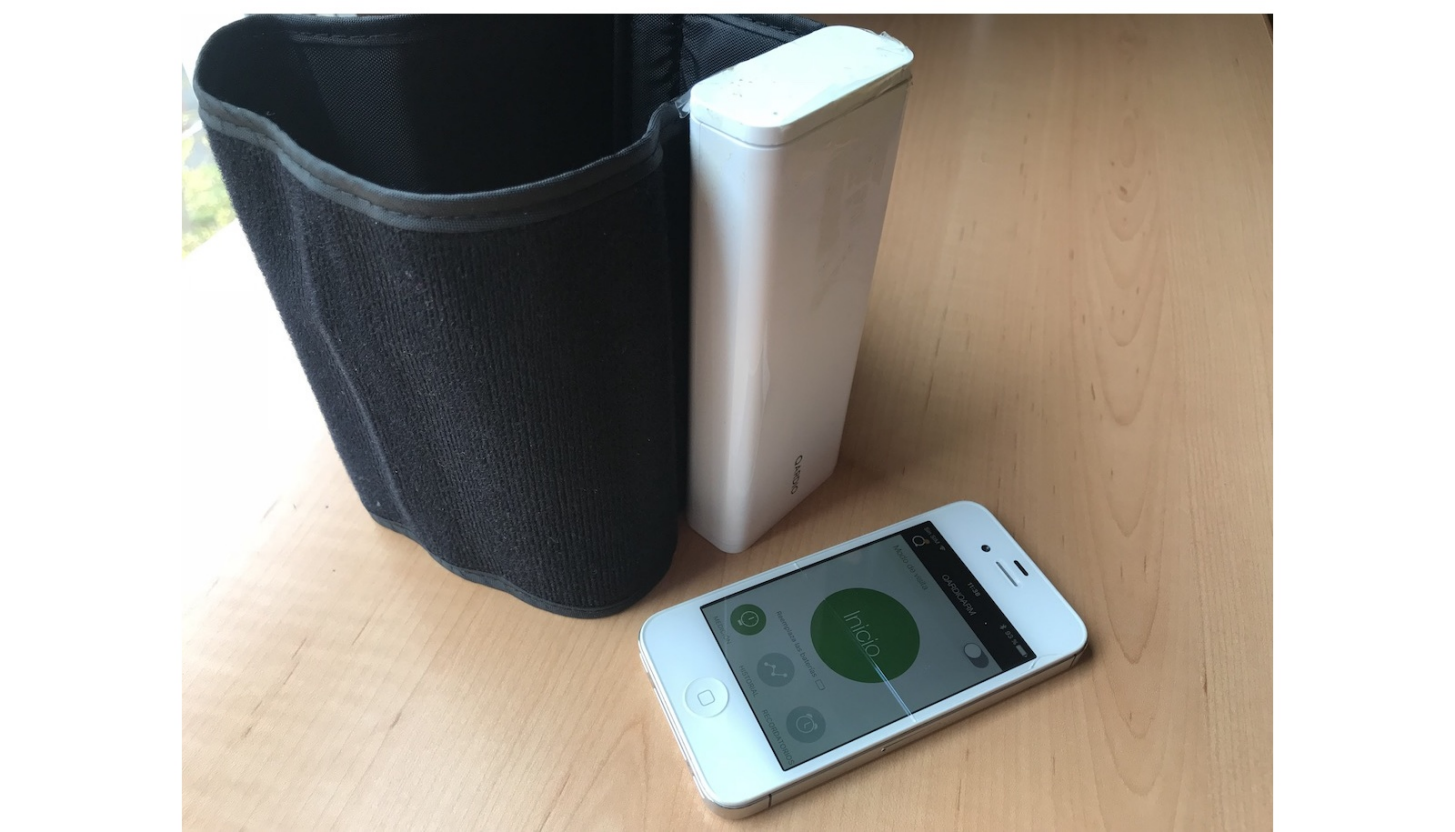
QardioArm is a device with a cuff used with an app for visualization of blood pressure known as Qardio.

Therefore, increased use of self-measured BP in the care of patients with arterial hypotension or hypertension could be beneficial. Recent clinical practice guidelines identified several indications for self-measured BP, which may provide for more standardized measurement of BP [5-17]. Today’s powerful mobile technologies can greatly facilitate self-measured BP by giving individuals more control over their health and well-being than conventional clinic-bound medical devices and allowing clinicians to evaluate the patient’s status and provide timely feedback remotely [18-28].

In a recent market study of mobile phone apps for managing hypertension, most were focused on health management. Only 14% of these Android and iPhone apps transformed a mobile phone into a BP measuring device [22]. QardioArm (Atten Electronic Co Ltd, Dongguan, China) is a device with a cuff used with an app for visualization known as Qardio [29] (Figure 1), and it may be a good alternative to enable self-measurement of BP by patients. None of these apps involved a BP cuff nor had any been validated against a gold standard. Only 3% of these apps were developed by health organizations, such as universities or professional organizations [18,20,22,23,25-28]. Nonetheless, consumers downloaded these apps for measuring BP and heart rate (HR) and evaluated them favorably, even though they had not been validated [22,24,26,30].

Self-measured BP devices are only useful and beneficial to the extent that they are user-friendly and accurate [6-9,11,13,14]. Self-measured BP devices should be validated by independent experts according to accepted protocols designed specifically for this purpose, such as those established by the British Hypertension Society [31], the Association for the Advancement of Medical Instrumentation [32], and the European Society of Hypertension (ESH) [33,34]. The ESH called for self-measured BP devices to be validated with the 2010 version of the ESH International Protocol [35]. Accordingly, we evaluated the accuracy of self-measured BP and HR measurements obtained with one such mobile app, the QardioArm, with the ESH International Protocol.

## Methods

We examined the concordance between two European Community-certified devices for measuring BP and HR in a study involving repeated measurements conducted in the city of Ciudad Real, Spain, between May and August 2016. The Clinical Research Ethics Committee of Hospital Clínico San Carlos in Madrid, Spain, approved this study (number 16/179-E). This study complies with the ethical principles of the Declaration of Helsinki [36], including amendments from 2000 to 2013.

### Participants

We recruited 125 participants from the general population of Ciudad Real using snowball sampling, from May to August 2016. A total of 100 participants completed the study; 25 participants did not return for the second measurement. According to the ESH International Protocol [35], a minimum of one-third of the total number of participants must be men and a minimum of one-third must be women. The participants satisfied the eligibility criteria in the latest revision of the ESH International Protocol [33-35], which are as follows:

Inclusion criteria:

1. Demographic: men and women aged at least 25 years. Of the total number of participants, at least one-third must be men and one-third must be women.
2. Temporal and geographic: healthy adult volunteers able to attend assessments in Ciudad Real.
3. Clinical: ideal BP range of 90 to 180 mm Hg for systolic blood pressure (SBP) and 40 to 130 mm Hg for diastolic blood pressure (DBP), and arm circumference of 220 to 320 mm.

Exclusion criteria:

1. Clinical: sustained arrhythmia, circulatory problems in which the use of a cuff is contraindicated, and/or pregnancy.
2. Cognitive impairment that leads to inability to follow instructions.

All participants gave their written informed consent to participate in this study.

Before recruitment, we calculated with the GRANMO sample size calculation program (Institut Hospital del Mar d’Investigacions Mèdiques, Barcelona, Spain) that a sample size of 125 would give 80% statistical power (α=.05) and two-tailed test to detect a difference equal to or greater than 0.9 mm Hg in BP. This calculation was based on the assumptions of a standard deviation of 3.2 mm Hg [37] and a loss to follow-up rate of 20%.

### Procedure

Participants visited the study site for two measurement sessions separated by 5 to 7 days. The first author (VMP) collected all the data. Both BP and HR were measured in the left arm. The central portion of the cuff was placed at heart level, with the bottom edge 1 to 2 cm above the elbow.

**Figure 2:**
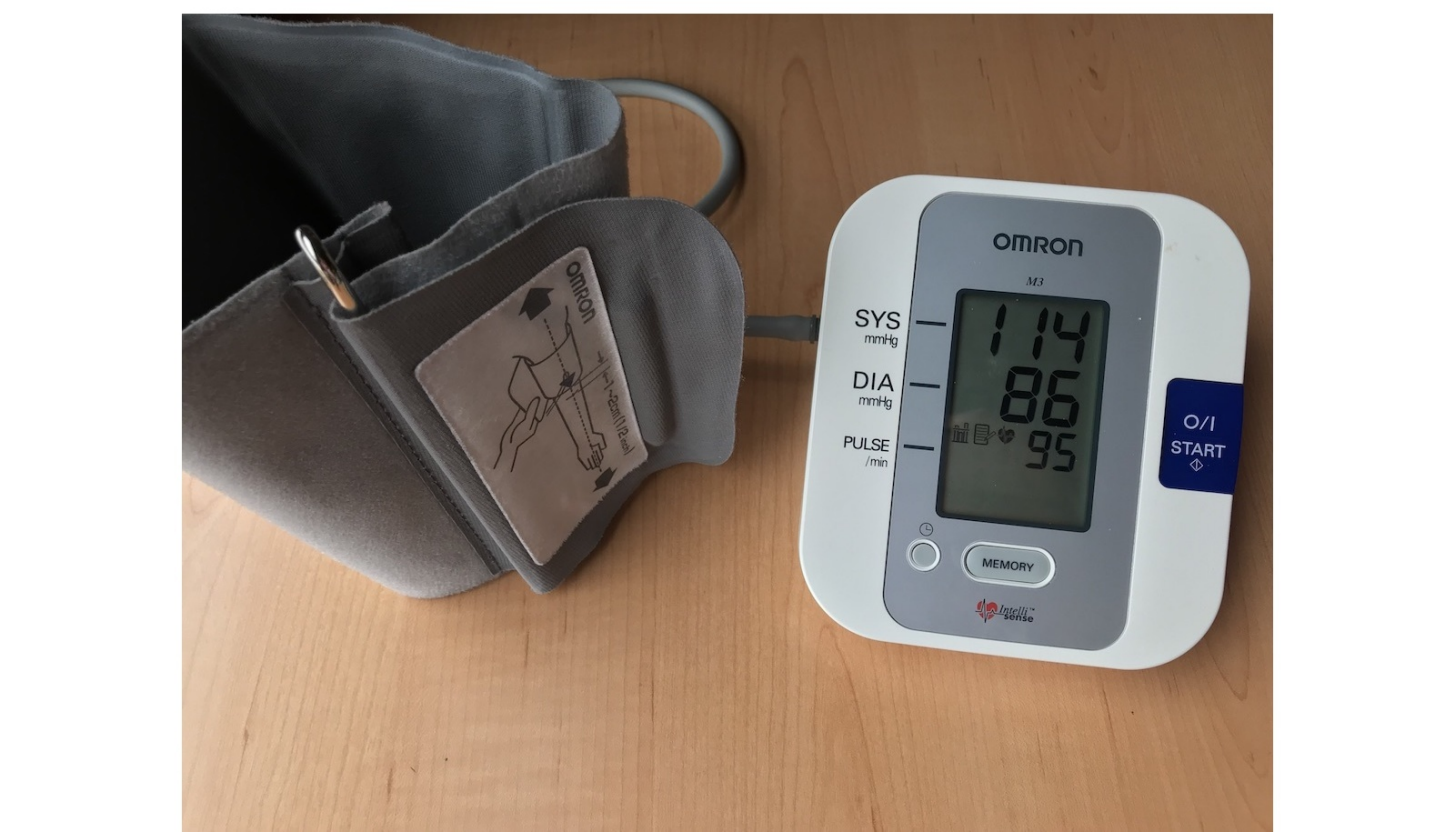
The Omron M3 blood pressure monitor.

During the first session, each participant reported his/her sex and date of birth. Measurements were taken of the participant’s weight (with an automatic digital scale), height (with a wall-mounted measuring rod), and arm circumference (with a tape measure at the midpoint between the acromion and olecranon). The participant then relaxed for 10 minutes before baseline BP and HR were measured with the criterion BP monitor Omron M3 (HEM-7200-E, Omrom Healthcare Co Kyoto, Japan) (Figure 2). Next, BP and HR were measured with the test device QardioArm only to confirm that the test device was working correctly on the participant (these were not included in analysis).

The rest of the first session involved seven BP and HR measurements, alternating between the criterion and test devices, with 2 to 3 minute intervals between measurements. The first, third, fifth, and seventh measurements were made with the criterion device; the second, fourth, and sixth measurements were made with the test device. The second measurement session involved the same series of seven measurements, with identical procedures carried out by the same researcher, which occurred on the same day of the week and at the same time after lunch, in the same room with identical conditions, as far as it was possible.

During measurements, participants were calm and quiet while sitting with their feet parallel and flat on the floor, their legs uncrossed, and their left hands resting palm side up on a flat surface. The room was maintained at an appropriate temperature and kept free from noises and distractions during the sessions [33,34].

Of the 125 participants who completed the first session, 100 also completed the second session. Our analysis focused on data from just these 100 participants.

### Study Devices

The Omron M3 Intellisense was the criterion device used as a benchmark in our study. It has CE 0197 certification and has been validated by the ESH between the Omron M3 and a different criterion device with mean 1.7 (SD 3.2) mm Hg for systolic and mean –0.9 (SD 2.6) mm Hg for diastolic [36].

The Omron M3 is a fully automatic BP monitor operating on the oscillometric principle. It has a range of 0 to 299 mm Hg for BP and 40 to 180 beats per minute (bpm) for HR. The cuff is inflated using an electric pump and deflated by means of a pressure release valve.

After each measurement, the SBP, DBP, and HR are shown on the Omron M3’s LCD screen. The device can also display a symbol on the screen indicating an irregular heartbeat, which is detected during measurement of SBP and DBP.

The QardioArm app for mobile phones and tablets was the test device in our study. It has CE 0734 certification and is a fully automatic, noninvasive wireless BP monitor for measuring SBP, DBP, and HR in adults. The QardioArm also operates on the oscillometric principle and has an inflatable cuff, which is placed around the upper arm. The cuff is inflated automatically and deflated using a controlled pressure release valve. The cuff is suitable for an arm circumference of between 22 and 37 cm. The measuring range is 40 to 250 mm Hg for BP and 40 to 200 bpm for HR.

The QardioArm has an automatic screen with graphics and visuals to facilitate interpretation of the data. The monitor connects to the free Qardio app on any device with Bluetooth 4.0 that runs iOS 7.0 or later or Android 4.4 or later. It can be used with iPhones, iPods, iPads, Apple Watches, and Android mobile phones and tablets. The data are stored on the mobile phone or tablet and therefore the pattern of values over time can be viewed. The app can be configured to issue reminders and warnings, and the measurements and progress can be shared in real time with other users *.*

The QardioArm weighs approximately 310 g without batteries and measures 6.8 cm in width, 3.8 cm in height, and 14 cm in length, with the cuff closed. It requires four AAA batteries.

### Statistical Analysis

All data were analyzed using SPSS version 19 (IBM Corp, Armonk, NY, USA). Statistical significance was set at *P*<.05.

We computed univariate summary statistics on participants’ characteristics. Body mass index (BMI) was defined as weight in kilograms divided by height (in meters) squared. To assess whether the BP and HR variables were normally distributed, we conducted one-sample Kolmogorov-Smirnov tests, applying the Lilliefors significance correction. Significant probability values in this test indicate nonnormality.

To evaluate the reliability of the QardioArm, we computed the intraclass correlation coefficient (ICC) for comparing measurements taken within the same session (intrasession) and in different sessions (intersession) [38]. The participants were the groups in each ICC analysis. Each intrasession evaluation involved the three measurements obtained using the test device for a particular primary variable (SBP, DBP, and HR). We used the session means of a variable in calculating the intersession ICC.

We interpreted ICC values with the guide proposed by Landis and Koch [39]: ≤0.20=slight agreement, 0.21-0.40=fair agreement, 0.41-0.60=moderate agreement, 0.61-0.80=substantial agreement, and ≥0.81=almost perfect agreement. We also followed the recommendations of Portney and Watkins [40], who suggested that an ICC of 0.90 or higher for clinical measurements indicates they are reliable.

We also calculated the standard error of measurement (SEM) for each intrasession and intersession primary variable. SEM values indicate the degree of error, with low values indicating low error. We used the SEM formula of the standard deviation multiplied by the square root of (1–ICC). To identify systematic error over time, we also compared first and second session means on each primary variable with paired-sample Student *t* tests.

To determine the validity of the QardioArm, we compared the QardioArm measurements with those made by the Omron M3, using both the ESH’s International Protocol for the validation of BP measuring devices and the Student *t* test. Following the ESH protocol [33,34], we computed the absolute values of the differences between successive pairs of the seven measurements in a session (second–first, second–third, fourth–third, fourth–fifth, sixth–fifth, and sixth–seventh). This gives three paired differences for each variable (SBP, DBP, and HR), participant, and session. We classified whether the paired differences were ≤5, ≤10, ≤15, or >15 mm Hg for BP and ≤3, ≤5, ≤8, or >8 bpm in the case of HR. The four levels of difference correspond to very accurate, slightly inaccurate, moderately inaccurate, and very inaccurate, respectively [33,34]. If the device passed during both sessions, it could be recommended for clinical use. If it did not pass during the second session, it could not be recommended for clinical use.

We compared the observed number of differences (out of 300: three paired differences for a variable × 100 participants in a session) falling into these categories with the standards specified in the ESH International Protocol. There were two phases of this comparison. If a test device did not pass during the first session, the validation process was terminated. If it passed during the first session, the second session was started.

In addition to following the ESH International Protocol, we computed Student *t* test for independent samples to check whether there were differences between devices in BP and HR measurements. For these tests, we used the mean values of the three study variables obtained from both sessions for the different devices. We also compared the mean intersession differences for the two devices. Furthermore, we calculated the Pearson correlations between the devices for the mean participant values on the three primary variables [41].

Finally, we produced Bland-Altman plots [42] to display the agreement between the two devices. These plots show the difference between each pair of measurements on the y-axis against the mean of each pair of measurements on the x-axis.

## Results

Table 1 shows the sociodemographic characteristics of the study participants (N=100). There were significant mean differences between men and women in weight (*P*<.001), height (*P*<.001), and BMI (*P*=.009), but no differences in age (*P*=.10) and arm circumference (*P*=.08). The Kolmogorov-Smirnov tests showed that SBP (*P*=.14), DBP (*P*=.20), HR (*P*=.20), as well as weight (*P*=.17) and BMI (*P*=.20) were normally distributed

Table 2 shows the intrasession and intersession reliability results for the QardioArm. In every case, reliability was high for SBP, DBP, and HR, and there was no evidence of systematic error from one session to the next.

Table 3 shows the validity results for the QardioArm, with the Omron M3 as the criterion. The QardioArm passed both sessions of the ESH validation process for SBP, DBP, and HR.

Table 4 shows the comparisons between the devices in terms of mean values on the primary variables as well as the Pearson correlations between them at the participant level. The two devices produced very similar mean BP and HR estimates. The differences in mean BP values for the QardioArm and Omron M3 were less than 2 mm Hg and the differences in mean HR values between devices were less than 0.1 bpm. All intrasession and intersession Pearson correlations were greater than .96.

**Figure 3:**
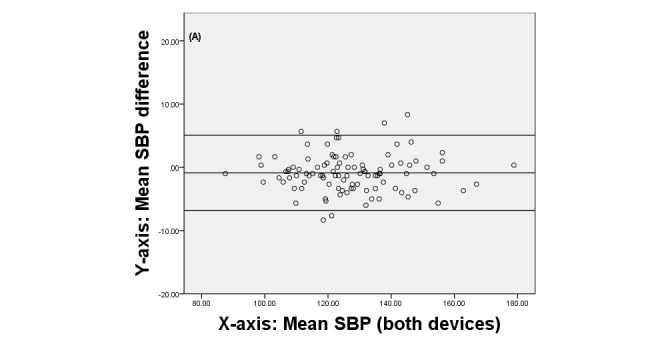
Bland-Altman plot comparing QardioArm and Omron devices for systolic blood pressure (SBP), for individual participants in session 1.

**Figure 4:**
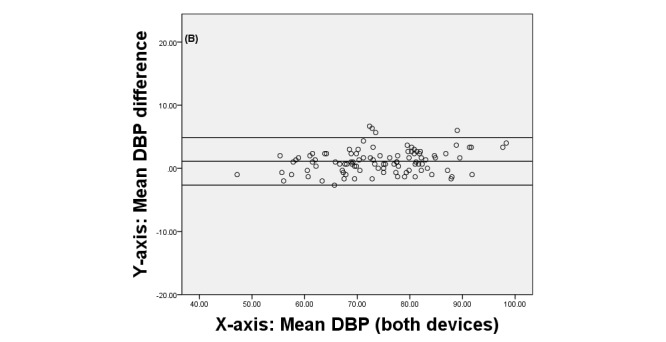
Bland-Altman plot comparing QardioArm and Omron devices for diastolic blood pressure (DBP) for individual participants in session 1.

**Figure 5:**
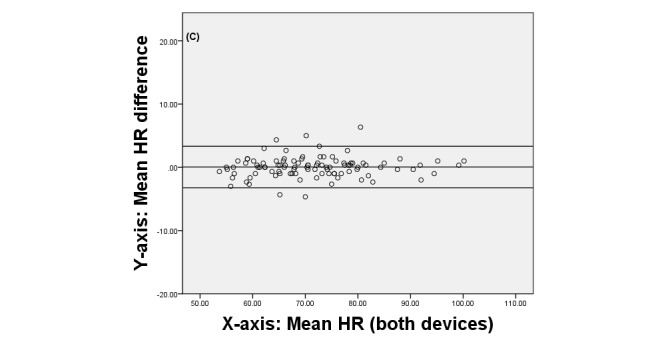
Bland-Altman plot comparing QardioArm and Omron devices for heart rate (HR) for individual participants in session 1.

Figures 3, 4, and 5 display the Brand-Altman plots for the BP and HR variables in the first session. For each variable and almost every participant, the difference between device means fell within the 95% confidence interval of all measurements. The plots for session 2 (not shown) gave very similar results.

**Table 1:** Sociodemographic and clinical characteristics of the sample.

Variables		Total group (N=100)		Male (n=37)			Female (n=63)		
		Mean (SD)	Range		Mean (SD)	Range		Mean (SD)	Range
Age (years)		52.2 (18.8)	25.0-89.0		52.3 (20.9)	25.0-89.0		52.2 (17.6)	25.0-83.0
Weight (kg)		76.2 (14.6)	49.5-125.1		84.6 (13.1)	65.3-125.1		71.3 (13.2)	49.5-102.4
Height (cm)		167.3 (8.1)	151.0-187.0		172.1 (7.1)	160.0-184.0		164.5 (7.4)	151.0-187.0
BMI^a^ (kg/m^2^)		27.1 (4.2)	18.9-38.2		28.5 (3.8)	22.2-38.2		26.3 (4.2)	18.9-37.7
Arm circumference (mm)		295.5 (35.2)	234.0-320.0		302.7 (33.9)	245.0-320.0		291.4 (35.6)	234.0-320.0
**Baseline SBP**^b^									
	Omron	130.9 (17.3)	88.0-180.0		136.0 (17.7)	101.0-180.0		127.9 (16.4)	88.0-167.0
	QardioArm	128.3 (17.2)	87.0-178.0		131.9 (17.4)	100.0-178.0		126.1 (16.8)	87.0-168.0
**Baseline DBP**^c^									
	Omron	74.2 (10.0)	49.0-98.0		73.5 (11.7)	57.0-98.0		74.6 (8.9)	49.0-97.0
	QardioArm	75.8 (10.8)	49.0-103.0		75.4 (12.3)	54.0-103.0		75.9 (9.9)	49.0-103.0

^a^BMI: body mass index.

^b^SBP: systolic blood pressure.

^c^DBP: diastolic blood pressure.

**Table 2:** Intrasession and intersession reliability for the QardioArm in sessions 1 and 2.

Variable		Mean (SD)		Range		ICC (95% CI)		*P* value		SEM
**Session 1**										
	Systolic pressure (mm Hg)		126.5 (16.1)		87.0-179.0		0.89 (0.84-0.92)			5.35
	Diastolic pressure (mm Hg)		74.8 (10.3)		46.7-100.3		0.91 (0.87-0.93)			3.15
	Heart rate (bpm)		71.4 (10.4)		53.3-100.7		0.92 (0.87-0.94)			3.00
**Session 2**										
	Systolic pressure (mm Hg)		124.9 (15.8)		89.7-171.7		0.91 (0.86-0.95)			4.67
	Diastolic pressure (mm Hg)		73.9 (9.6)		51.7-95.7		0.87 (0.82-0.90)			3.52
	Heart rate (bpm)		70.1 (11.0)		44.3-96.0		0.95 (0.93-0.96)			2.53
**Intersession**										
	Systolic pressure (mm Hg)		125.7 (15.3)		87.0-179.0		0.91 (0.87-0.94)		.09	4.73
	Diastolic pressure (mm Hg)		74.3 (9.4)		46.7-100.3		(0.83-0.92)		.17	3.36
	Heart rate (bpm)		70.7 (9.8)		44.3-100.7		0.80 (0.70-0.86)		.14	4.82

**Table 3:** shows the validity results for the QardioArm, with the Omron M3 as the criterion. The QardioArm passed both sessions of the ESH validation process for SBP, DBP, and HR.

				Category										Result	Mean difference ± SD^a^
				˂5 mm Hg/ 3 bpm^b^		˂ 10 mm Hg/ 5 bpm^b^		˂ 15 mm Hg/ 8 bpm^b^		2/3 within 5 mm Hg/3 bpm^c^		0/3 within 5 mm Hg/3 bpm^c^			
**Session 1**															
	**Required**														
		Two of^d^	219		261		288		N/A^e^		N/A		N/A		N/A
		All of^f^	195		243		279		N/A		N/A		N/A		N/A
	**Observed**														
		SBP1^g,h^	242		286		298		N/A		N/A		Pass		3.43±3.13
		DBP^i^1	277		295		299		N/A		N/A		Pass		2.43±2.35
		HR^j^1	265		287		297		N/A		N/A		Pass		1.60±1.92
		SBP2^k^	256		289		300		N/A		N/A		Pass		3.29± 2.74
		DBP2	266		295		298		N/A		N/A		Pass		2.79±2.86
		HR2	272		287		295		N/A		N/A		Pass		1.61±1.99
**Session 2**															
	Required		N/A		N/A		N/A		≥ 72^l^		≤ 9^m^		N/A		N/A
	**Observed**														
		SBP1	N/A		N/A		N/A		87		0		Pass		N/A
		DBP1	N/A		N/A		N/A		95		0		Pass		N/A
		HR1	N/A		N/A		N/A		90		0		Pass		N/A
		SBP2	N/A		N/A		N/A		94		0		Pass		N/A
		DBP2	N/A		N/A		N/A		95		2		Pass		N/A
		HR2	N/A		N/A		N/A		93		0		Pass		N/A

^a^SD: standard deviation.

^b^Frequencies of paired measurements (of 300) within particular ranges.

^c^Frequencies of participants (of 100) with measurements meeting certain criteria.

^d^“Two of” means that two of the three conditions given in the same row must be met.

^e^N/A: not applicable.

^f^“All of” means that all three requirements given in the row must be met.

^g^SBP: systolic blood pressure.

^h^Session 1.

^i^DBP: diastolic blood pressure.

^j^HR: heart rate.

^k^Session 2.

^l^At least two of the three pairs of differences for at least 72 participants must fall in the 5 mmHg category.

^m^The three pairs of differences of no more than 9 participants may fall outside the 5 mmHg category.

**Table 4:** Comparison of Qardioarm and Omron devices in mean estimates and correlations of individual means between devices.

Variable		Omron, mean (SD^a^)	QardioArm, mean (SD)	t-test *P* value	*r*	*P* value
**Intrasession 1**						
	Systolic pressure (mm Hg)	127.34 (16.02)	126.47 (16.06)	.70	.983	<.001
	Diastolic pressure (mm Hg)	73.66 (9.82)	74.78 (10.29)	.43	.984	<.001
	Heart rate (bpm)	71.31 (10.29)	71.36 (10.42)	.97	.987	<.001
**Intrasession 2**						
	Systolic pressure (mm Hg)	125.65 (16.02)	124.96 (15.84)	.76	.984	<.001
	Diastolic pressure (mm Hg)	72.23 (9.08)	73.90 (9.61)	.21	.966	<.001
	Heart rate (bpm)	70.12 (10.65)	70.05 (10.99)	.96	.987	<.001
**Intersession**						
	Systolic pressure (mm Hg)	126.50 (15.47)	125.71 (15.30)	.72	.991	<.001
	Diastolic pressure (mm Hg)	72.95 (8.90)	74.34 (9.43)	.29	.982	<.001
	Heart rate (bpm)	70.72 (9.68)	70.70 (9.76)	.99	.993	<.001

^a^SD: standard deviation.

## Discussion

The QardioArm is recommended by dabl Educational Trust, which is an organization that provides an interpreted one-page BP report for the medical team and patient [43], but there are not any validation studies on the QardioArm published in a peer-reviewed journal. Recently, a study compared QardioArm with three other mobile phone-compatible BP measuring devices using a handheld aneroid sphygmomanometer as the reference device [44], but the study did not follow the ESH International Protocol.

We evaluated the reliability and validity of the QardioArm device for self-measured BP with 100 participants in two measurement sessions. The QardioArm displayed very consistent readings both within and across sessions. The QardioArm measurements corresponded closely to those from the previously validated criterion device, the Omron M3. The QardioArm easily passed all validation standards set by the ESH International Protocol. Therefore, the QardioArm can be recommended for clinical use in individuals with similar characteristics to those who participated in this study, such as adults aged 48 to 56 years with a BMI between 25 and 29, not pregnant, and without cardiac arrhythmia, vascular problems, or arteriovenous fistulas in the arm.

There are several limitations of the ESH International Protocol and, thus, our study. The ESH International Protocol uses the same validation requirements (eg, ≤5 mm Hg difference indicating accuracy) for both SBP and DBP, even though the magnitude of diastolic values is usually half that of systolic measurements.

Also, HR as a parameter is not considered in any version of the ESH International Protocol despite the fact that automatic sphygmomanometers produce measurements of HR as well as BP. We have not found any studies of HR validation attempts in the literature that follow the ESH International Protocol [37,45-49]. Therefore, ours may be the first study to validate this parameter with the criteria of the ESH protocol for BP. We set the requirements for HR validation to be roughly proportional to the magnitude of the measurements, and they may even be more demanding than the BP requirements of the ESH International Protocol.

Moreover, the recommendations made in the ESH International Protocol regarding the populations to which validation results can be applied may not be followed faithfully in clinical practice. The ESH International Protocol imposes certain gender requirements and limits validation studies to individuals older than 25 years who have BPs and arm circumferences within specific ranges. Because these subgroups represent only a portion of the large heterogeneous population with BP abnormalities, extrapolation of ESH validation results to other specific populations could be considered unsafe. Additional validation studies are needed if the product is to be used in other subpopulations, such as pregnant women, obese individuals, children, or individuals with particular conditions, such as arrhythmia.

The ESH International Protocol also does not indicate the number of validation studies needed to confirm the accuracy of the device. According to experts, at least two validation studies should be performed in different centers and in different populations [37]. The protocol of the Association for the Advancement of Medical Instrumentation recommends more than one study, but does not specify the number of studies or devices [32]. Because the QardioArm device has not been validated previously, we are unable to compare our findings with those of other authors. When compared with the Omron M3, the QardioArm tends to give very slightly and nonsignificantly lower SBP and very slightly and nonsignificantly higher DBP readings. We recommend that the QardioArm be further validated with different study designs and study sites and with different types of populations.

There is a high concordance between the measurements made with QardioArm, both intrasession and intersession. The QardioArm mobile app has validity and there is a direct linear correlation between QardioArm measurements and the previously validated Omron M3 measurements, so it can be used without the risk of major measurement error.

## Abbreviations

BMI: body mass index
BP: blood pressure
DBP: diastolic blood pressure
ESH: European Society of Hypertension
HR: heart rate
ICC: intraclass correlation coefficient
SBP: systolic blood pressure
SEM: standard error of measurement
